# What impact do personality traits have on self-perception of dental aesthetics?

**DOI:** 10.1186/s13005-023-00358-1

**Published:** 2023-03-15

**Authors:** Qian Xu, Wulong Du, Feiou Lin

**Affiliations:** 1 Department of Orthodontics, School and Hospital of Stomatology, Wenzhou Medical University, Wenzhou, 325000 China; 2 Department of Stomatology, Lishui Central Hospital and Fifth Affiliated Hospital of Wenzhou Medical College, Lishui, 323000 China

**Keywords:** Orthodontics, Psychosocial impact of dental aesthetics questionnaire, Eysenck personality, Oral health-related quality of life

## Abstract

**Background:**

Malocclusion has significant social, psychological and physical impacts on the quality of life. This study aimed to study psychosocial impact of dental aesthetics among students, and how it was modified by personality traits.

**Methods:**

Chinese version of Eysenck Personality Questionnaire Short Scale of (EPQ-RSC) and Chinese version of Psychosocial Impact of Dental Aesthetics Questionnaire (PIDAQ) were used to investigate 555 undergraduate students, aged 18 to 24 years. The participants and investigators self-rated their own dental aesthetics using the Aesthetic Component (AC) of the Index of Orthodontic Treatment Need (IOTN). The malocclusion was assessed by two independent investigators using the Dental Health Component (DHC) of IOTN.

**Results:**

No statistically significant difference was found for gender (*p* = 0.829) nor for majors (*p* = 0.598) in the psychosocial impact of dental aesthetics. Total and subscale PIDAQ scores differed significantly among the 3 IOTN grades. PIDAQ scores strongly correlated with Eysenck personality. The dimensions of extraversion and neuroticism, and dental health component grades had significant impact on participants' psychosocial well-being of dental aesthetics.

**Conclusions:**

Neurotic people with high self-concerns perceived greater psychosocial impact. Outgoing people appear to be less affected psychosocially by dental aesthetics.

## Introduction

Malocclusion is a misalignment of teeth, malformation and/ or ill proportion of maxillofacial structures, which can be found in 72.92% of the Chinese population. Although malocclusion is neither conventionally accepted as a disease nor treated by professional as a life-threatening condition, it has significant social, psychological and physical impacts on the quality of life, which is thought to worsen with the increased severity of misalignment [1, 2]. In line with the quality of life assessment defined by World Health Organization, dental health means not only the absence of oral dysfunction, but also positive dental-related psychosocial life and self-contentment [3]. Meanwhile, need for orthodontic treatment shows a continuous increase in dental care services [1, 4].

The need for orthodontic treatment can be measured by several techniques including Dental Aesthetic Index, Handicapping Labial-lingual Deviation with California Modification, and Index of Orthodontic Treatment Need (IOTN). Among them, IOTN provides much more accurate measurement with rapid implementation [5], because IOTN measures both requirements on the Dental Health Component(DHC) as well as the Aesthetic Component(AC).

However, none of those above mentioned traditional techniques include quantitative or qualitative factors of quality of life, particularly in terms of functional and psychosocial well-being, into the assessments [6]. Oral Health-related Quality of life (OHrQol), a multidimensional construct to evaluate dental treatment needs and outcomes, is therefore introduced to meet the criteria of health [7]. Yet few of its indexes has been specially developed for orthodontics. Psychosocial Impact of Dental Aesthetics Questionnaire (PIDAQ), a specialized tool for malocclusion, is widely used to assess the dental aesthetic related psychosocial impact, as it provides measurements on the orthodontic-related quality of life and psychometric results [8]. Unexpectedly, some patients have been found suffering, disproportional for the mildness of malocclusion. This may be a result of individual personalities affecting their aesthetic attitudes [9]. Furthermore, researchers in both cosmetic and orthognathic specialties found better accepted treatment outcomes when psychological characteristics of the patients were taken into account during treatment planning [10]. So it is also necessary to consider personality into evaluations in addition to OHrQoL.

The Eysenck Personality Questionnaire(EPQ) has been widely applied and revised in many countries to study personality traits.As reported, both negative and positive personality traits could be attributed to dental appearance [11]. For example, negative inclinations are always associated with passive self-assessment of dental appearance [2, 12]. Personality traits that are significantly correlated with malocclusion can be built in EPQ [13]. Jiang et al. found that personality had significant impact on the stress system, particularly the cognitive appraisal and negative coping factors [14]. Components including personality, life event, social support and cognitive appraisal interrelate and regulate each other, which eventually determine practical coping. From an orthodontic perspective, these determine patient's desire towards the treatment and coping strategy during orthodontic care. However a few have presented the influence of personality traits on the relationship between self-perceived malocclusion and its psychosocial impacts. This is also the motivation behind present study.

Knowing oneself and knowing the enemy can win a hundred battles. The objective of this study was to investigate psychosocial impact of undergraduate Chinese students’ dental aesthetics through completing their PIDAQ, EPQ and self-rating of own dental aesthetics, how it was modified by individual personality, and participants are grouped by IOTN. This study will help to outline participants’ perception of their oral aesthetics and orthodontic treatment, and thereby to understand patient expectations in advance, enable a better planned dental treatment, and make patients mostly satisfied.

## Material and methods

### Ethical aspects and sample

In this cross-sectional study, ethical approval was obtained from the Health Research Ethics Board of Wenzhou Medical University (WYKQ2018005). Informed consents were provided by all participants.

Participants in this cross-sectional research were undergraduates aged 18 – 24 years from two universities in Wenzhou, People’s Republic of China, and the period of participants’ recruitment is between 2021/03/01 to 2021/6/30. A random selection program designed by Microsoft Visual Basic (Microsoft, Redmond, Wash) was conducted among dormitories to select and recruit participants, finally 125 dormitories were selected, and a total of 555 participants were recruited for the study. Data from 482 participants were used in the final analysis. The dropout rate is 13.15%. Participants with the following conditions were excluded from the study: (1) craniofacial anomalies such as cleft lip, cleft palate, and traumata, (2) severe skeletal discrepancies requiring orthognathic surgery, (3) missing teeth or implants, (4) previous or undergoing orthodontic assessments, (5) refusal to dental examinations, and (6) missing answers in one of the two questionnaires resulted in the exclusion of the participant. These criteria were established to prevent potential confounding effects of such conditions on participants’ OHRQoL, as well as to assure the validity and accuracy of our research. Participants were informed of the study purpose from the start, and of the procedures accordance to medical article ethics guidelines. They were also assured of the confidentiality of the information collected. In addition, dental students were included for comparison.

### Study design, setting and questionnaire

The participants were asked to complete the PIDAQ, the short scale of EPQ, and self-rating of own dental aesthetics. Investigators made separate ratings on participants’ aesthetics and examined the participants’ occlusion using the modified Dental Health Component as elaborated below.

### Index of orthodontic treatment need (IOTN)

The requirement of treatment was assessed according to IOTN, which consisted of DHC and AC. Dental aesthetics were scored by both the investigators and the participants, where IOTN-AC was used to measure the cognition disparity between professionals and laypeople. From 10 black and white photographs of anterior teeth showing different degrees of malocclusion, participants were asked to indicate the photograph that resembled their dental appearance the most. Then oral examinations were performed by investigators and the dentition of each participant was scored with IOTN-AC scale as well as IOTN-DHC to determine the highest scoring anomaly. Intra-oral examinations were performed independently by two investigators who had been trained and calibrated (inter- and intra-examiner reliability: weighted kappa 0.83 and 0.92, respectively). All research subjects were measured twice by the same researcher at 2-week intervals, and the average of two measurements was analyzed. The classifications were as follows: DHC grades 1–2 and AC grades 1–4 = no or slight need for orthodontic treatment; DHC grades 3 and AC grades 5–7 = moderate or borderline need; DHC grades 4-5and AC grades 8–10 = definite need [15]. Classification in other studies can be slightly different. For example, in the NHS, AC is used for borderline cases with DHC Grade 3 and if the case has a high AC score (usually above 6), NHS treatment is needed.

### Psychosocial impact of dental aesthetics questionnaire (PIDAQ)

PIDAQ is a psychometric technique to assess orthodontic-specific aspects of life quality. The Chinese version of PIDAQ has been tested for its validity, reliability, and factorial stability [16]. Invented by Klages initially, the final version is composed of eight items about social impact, six items about psychological impact, three items about esthetic concern, and six items about dental confidence. A five-point Likert scale was applied to rate these items. The response options were defined as follows: 0 = not at all; 1 = a little; 2 = somewhat; 3 = strongly; 4 = very strongly. A reversed dental confidence (DC) was adapted to keep the scoring consistent in present study. A higher PIDAQ rate meant a greater degree of negative psychosocial impact. In addition, a respondent questionnaire with one unanswered item was treated as mean imputation, while those with 2 or more deficient answers were regarded invalid.

### Eysenck personality questionnaire -short scale of Chinese version(EPQ-RSC)

Eysenck Personality Questionnaire(EPQ), one of the personality inventories developed by Eysenck, distinguishes itself with less concept, convenient investigation, high reliability and validity compared to other personality questionnaires compiled by factor analysis [17]. The Short Scale of Chinese version of EPQ(EPQ-RSC), which has been proved to be more applicable in China than the full version, was used to assess the personality of the respondents. It consisted of 48 questions with yes and no options, and included four factors of extroversion/introversion (E: representing sociability, liveliness, and openness), neuroticism (N: emotional instability and anxiousness), psychoticism (P: tough-mindedness, aggressiveness and indifference) and lie (L: unsophisticated dissimulation and social naivety or conformity). Raw score of each dimension was transformed into T-score, where high scores suggested an outgoing, neurotic or stubborn personality. The corresponding inclinations were defined as following: 43.3–56.7 = ambiversion, 38.5–43.3 and 56.7–61.5 = tendentious type, < 38.5or > 61.5 = typical type. One invalid personality questionnaire with missing items was excluded from the study.

### Statistical analysis

The Statistical Package for Social Sciences (SPSS, version 18.0; Chicago, IL, USA) was applied to calculate data and to run one-way analysis of variance (ANOVA), with least significant difference post hoc test to assess PIDAQ scores across groups of different IOTN. The data was the normal distribution by Skewness test. The PIDAQ and EPQ-RSC variables between groups were compared with the Mann–Whitney U test and t-test. The strengths of linear relationships between pairs of variables were assessed by Pearson and Spearman correlation analysis. EPQ personality effect (four factors) was analyzed by generalized multiple linear regression. The level of significance was set at *p* < 0.05 and high significance at *p* < 0.01.

## Results

**Fig. 1:**
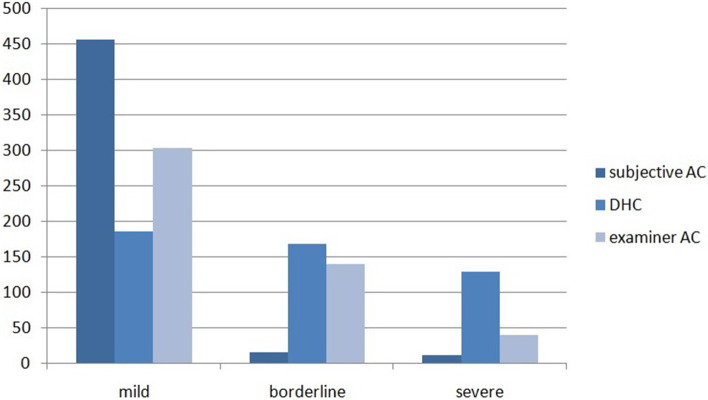
Respondents distribution classified by indices of treatment need (IOTN)

Table 1 showed the demographics of all 482 participants, including 205 males and 277 females with the mean age of 21.24 ± 1.4 years. Additionally, education background has also been recorded. There were 80 dental students and 402 students majoring in other disciplines. All the numerical data were normally distributed except for four sub-items of PIDAQ. Clearly, Chi-square test did not show significant differences between genders (*p* = 0.829) or between majors (*p* = 0.598) in relation to the PIDAQ scores and perception deviation. IOTN results of participants are also listed in Table 1 and illustrated in Fig. 1. As shown in Table 2, we compared PIDAQ statistics of three kinds of IOTN groups between two genders. Slight discrimination (Dental Confidence) could be noticed between genders, which lied in sub-items in a given situation (Table 2).

**Table 1:** Demographics of the participants

Demographic	Number	Percentage (%)	*p-*value (Chi-square/ one way ANOVA)
**Age (y)**			0.758
** < 20**	141	29.25	
** ≥ 20**	341	70.75	
**Gender**			0.829
** Male**	205	42.53	
** Female**	277	57.47	
**Major**			0.598
** Dental**	80	16.60	
** Not dental**	402	83.40	
**Index of orthodontic treatment (IOTN-DHC)**			< 0.001^**^
** Mild /no (1–2)**	185	38.38	
** Moderate (3)**	168	34.85	
** Severe (4–5)**	129	26.76	
**Self-perception of dental aesthetics (IOTN-AC)**			0.009^**^
** Slight (1–4)**	456	94.61	
** Borderline (5–7)**	15	3.11	
** Definite (8–10)**	11	2.28	
Total	482	100	

*IOTN-DHC* Index of Orthodontic Treatment Need- Dental Health Component, *IOTN-AC* Index of Orthodontic Treatment Need- Aesthetic Component

^*^p < 0.05

^**^ *p* < 0.01

**Table 2:** The statistics comparison between genders sorted by three IOTN groups

	**No or slight need(*N* = 185)**			**Moderate need(*N* = 168)**			**Definite need(*N* = 129)**		
	**Male**	**Female**	***p***	**Male**	**Female**	***p***	**Male**	**Female**	***p***
**PIDAQ **^**a**^	24.81 (8.85)	22.91 (10.58)	0.204	28.49 (11.37)	30.41 (12.14)	0.300	29.82 (11.15)	30.84 (12.11)	0.619
**PIDAQ-Social Impact **^**b**^	3.03 (3.41)	2.87 (3.86)	0.291	4.12 (4.76)	4.53 (4.32)	0.333	4.09 (3.87)	4.57 (4.72)	0.792
**PIDAQ-Psychological Impact **^**b**^	4.19 (3.56)	4.62 (3.49)	0.291	5.08 (3.91)	6.04 (4.33)	0.159	5.07 (4.01)	6.47 (3.92)	0.036
**PIDAQ-Aesthetic Concern **^**b**^	0.97 (1.61)	0.88 (1.70)	0.355	1.59 (2.36)	1.62 (2.40)	0.889	1.64 (2.25)	1.64 (2.34)	0.984
**PIDAQ-Dental Confidence **^**b**^	16.6 (4.99)	14.5 (6.09)	0.028*	17.70 (5.22)	18.22 (5.31)	0.311	19.02 (5.22)	18.16 (4.89)	0.204
**Perception Deviation **^**b**^	1.55 (1.11)	1.35 (0.95)	0.636	2.36 (1.90)	1.96 (1.17)	0.338	2.88 (1.82)	2.64 (1.98)	0.294

Values are presented as mean (standard deviation)

*IOTN* Index of Orthodontic Treatment Need, *PIDAQ* Psychosocial Impact of Dental Aesthetics Questionnaire; Perception Deviation: deviation between subjective and objective IOTN-AC (Aesthetic Component of the IOTN.)

^a^t-test

^b^Mann-Whitney U-test

^*^p < 0.05

^**^ *p* < 0.01

Table 3 summarized the correlations between PIDAQ and the IOTN grades as well as Eysenck personality inclinations. The total and subscale PIDAQ scores correlated closely with both IOTN-DHC and IOTN-AC. Moreover, some parts of the personality traits, especially extroversion and neuroticism, were related with the psychosocial impact of dental aesthetics (*p* < 0.01).

**Table 3:** Correlation between PIDAQ scores and other variables(*N* = 482)

	**P*IDAQ total***	**SI**	**PI**	**AC**	**DC**
**DHC**	0.259^**^	0.150^**^	0.149^**^	0.145^**^	0.255^**^
**Objective AC**	0.268^**^	0.168^**^	0.137^**^	0.151^**^	0.264^**^
**Subjective AC**	0.379^**^	0.201^**^	0.236^**^	0.251^**^	0.387^**^
**T score of P**	-0.016	0.023	0.001	0.058	-0.073
**T score of E**	-0.227^**^	-0.118^**^	-0.140^**^	-0.166^**^	-0.217^**^
**T score of N**	0.221^**^	0.256^**^	0.237^**^	0.240^**^	0.004
T score of L	-0.018	-0.052	-0.058	0.010	0.039

*PIDAQ* Psychosocial Impact of Dental Aesthetics Questionnaire, *SI* social impact, *PI* psychological impact, *AC* aesthetic concern, *DC* Dental Confidence, *DHC* Dental Health Component of IONT, *P* psychoticism, *E* extroversion/introversion, *N* neuroticism, *L* lie

^*^p < 0.05

^**^ *p* < 0.01

Generalized multiple linear regression of personality factors for PIDAQ showed a linear relationship between the parameters (Table 4), and extroversion and neuroticism significantly impact one's PIDAQ. Psychosocial influence changed almost linearly as the trait of personality inclined in predicted curves. Extraversion worked negatively with PIDAQ, while neuroticism behaved in the opposite manner. Other personality factors had no impact on PIDAQ statistically. Meanwhile, DHC and subjective perception of dental aesthetics contributed to personal cognition of psychosocial influence.

**Table 4:** Results of generalized multiple linear regression of factors for psychosocial impact of dental aesthetics (*N* = 482)

Coefficients ^a^				
**Parameter**	**B**	**Std. Error**	**t Value**	***p*-value**
**constant**	18.28^**^	4.50	5.22	< .001
**DHC**	3.43^**^	0.93	3.69	< .001
**Objective AC**	-0.15	1.16	-0.13	n.s
**Subjective AC**	2.93^*^	1.42	2.07	0.039
**T score of P**	-0.91	0.51	-1.78	n.s
**T score of E**	-1.37^**^	0.40	-3.42	< .001
**T score of N**	1.85^**^	0.43	4.33	< .001
T score of L	0.18	0.45	0.39	n.s

^a^ Dependent Variable: PIDAQ; *DHC* Dental Health Component of IONT, *AC* aesthetic concern, *P* psychoticism, *E* extroversion/introversion, *N* neuroticism, *L* lie

^*^p < 0.05

^**^ *p* < 0.01

## Discussion

As the quality of life improves, aesthetics requires much more attention than ever before. This can be seen in the dental field accordingly, where a significant increase in patients requiring orthodontic treatment has been witnessed. Certain psychological characteristics found in people with malocclusion could lead to internal insecurities which in turn may cause improper external relationship with others [13]. Therefore, orthodontists should adequately comprehend psychological health and personality inclination of patients regularly.

Here, we intended to achieve a certain level of understanding of the crowd psychology, which is very valuable in dental-related psychosocial cognition and might ultimately impact orthodontic treatment design. We used PIDAQ and EPQ to study the psychosocial impact of dental aesthetics, and how it was modified by individual personality.

This study targets at young adults (university students)because it is likely more accurate to analyze their self-perceived psychosocial impact, as they have already possessed a certain emotional stability and a more realistic view of dental-facial aesthetics [18]. Furthermore, young adults have a more stable self-concept than adolescents, and are more concerned about physical appearance than older individuals [8]. Other factors, including educational degree, marital status and income could also impact on the perception of dental aesthetics. University students were therefore selected in order to minimize the interference of demographics. Unlike previous researchers, we targeted young adults instead of patients. Most patients in an orthodontic clinic have had the intention for dental treatment, which may not represent the general public. However, the lack of investigation into the general public is a limitation of this study and further researches should be made to survey the public.

Table 1 showed the distribution of participants classified by IOTN grades and indicated that more than half of the participants have dental misalignment of various degree (IOTN-DHC met the borderline or definite demand). Several studies have reported frequency distribution skewed to the attractive (low) end in both examiner and subject AC scores [15]. Moreover, the skewness of objective aesthetic concern was less significant than the subjective one (Fig. 1). This abnormal distribution might indicate that both the laypeople and orthodontists tend to perceive malocclusions (AC) as more aesthetically pleasing than the normative treatment need (DHC) would indicate. This phenomenon adequately reflects that aesthetic consideration is the main reason for orthodontics nowadays. However, the instrument of IOTN was not perfect because AC did not feature: malocclusion of class III and class II, hypodontia, malformed teeth etc. These conditions were recorded as the most similar ones in the present study.

Perception difference between genders is an interesting topic of aesthetics. The present study showed no significant disparity in cognition or in the psychosocial impact of dental aesthetics except for a slight difference in the subordinate item (Table 2). Traditional opinions verify the gender difference in personality, and regard females as more anxious, sensitive and concerned about appearance [13]. Psychosocially, they may suffer more because of severe malocclusion, but even slight misalignment could affect their confidence. However, existing evidence about the influence of gender on self-perceived orthodontic need and psychosocial impact of dental esthetics is inconsistent. Some researchers have demonstrated the same result of slight or no gender difference as we did [19]. While others found higher needs for females or even the opposite result [20]. The unbalanced gender distribution was a potential limitation of this study. The importance of culture in shaping these gender differences could be further investigated [21]. Dental students were always ruled out in previous studies, because dental education endowed them with professional acknowledgement of oral aesthetic assessment. According to the present result, we found that students majoring in dentistry did not have a more precise perception than the others, which was interesting but not surprising. The reason for this phenomenon may be owed to the fact that Chinese dental students acquire professional knowledge of orthodontics only when they reach their senior year. The proportion of students majoring in dentistry is not high, so there are certain deficiencies and further research is needed. It is concluded that the lack of specialized study may be the cause of the indiscrimination.

It is believed that malocclusion exerts great influence on quality of life nowadays, which worsens with increased severity of malocclusion [22]. This is in close agreement with the results shown in Table 3, and the Spearman correlation coefficients indicated the main predictor of PIDAQ was self-perception of dental appearance. Moreover, all PIDAQ domains were scored variously in each type of malocclusion [23]. However, clinical experience and other studies indicated that some patients were under critical psychosocial impacts even due to mild malocclusion [24]. It was also observed in the present study that a few participants whose malocclusion required orthodontic treatment did not suffer as much as assumed by the authors. This phenomenon has been attributed to physical factors, but inherent factors including personality traits have always been ignored in orthodontics. McManus et al. have confirmed that aesthetic attitudes are strongly related to some particular psychological characteristics [9]. Psychologists from China also have pointed out that personality is the core of the mental impress system, in which perception plays an important role [14]. Clearly, self-perceptions of oral condition impact well-being in different ways along with the psychological status. These may provide a new direction for orthodontic and dental-aesthetic field [25].

We applied EPQ-RSC to assess participants' character inclination. No discrimination of personality was noticed between genders, and dimensions including extroversion and neuroticism were significantly related with the psychosocial impact of dental aesthetics (*p* < 0.01). These results indicated that extroversion negatively related with psychosocial impact while neuroticism acted positively. Neurotic people were more vulnerable to psychosocial impact than extroverted people. Multiple linear regression analyses in Table 4 illustrated this phenomenon with more precision. High score in extraversion was characterized by being outgoing, talkative, adventuresome, high on positive affect (feeling good), and suggested the need of external stimulation. High marks in neuroticism represented neurotic people,who had low activation thresholds, low ability to inhibit or control emotional reactions, and the tendency to experience negative affection in the face of very minor stressors [26]. Therefore, the psychosocial impact is less severe on a more outgoing person, but more heavily on a more emotionally unstable individual. Neither of the other two aspects (psychoticism and lie) correlates with psychosocial impact of aesthetics. This may be due to the monodirectional scales of these dimensions, which limits the correlation between personality and psychosocial influence. Psychoticism is associated with the liability to have a psychotic episode (or break with reality), which contributes for an early diagnosis of psychiatric disorders. Lie, the independent dimension to measure the dissimulation of the respondent, is the validity scale [26]. Strong relationship between personality and PIDAQ sub-domains were noticed in the moderate scale. This may provide a new direction in clinical research focused on the controversial stage where the misalignment meets the very borderline for orthodontic care and perceptions can be complicated.

The four sub-domains of PIDAQ include items referring to latent unpleasant social issues, inferiority complexes, dislike of dental appearance and impacts on personal pride respectively [8]. The explicit relationship between neuroticism and the factors confirms that neurotic people, compared to extroverts, are much more anxious and tense in situations of oral aesthetics, especially in case of critical deformity.

Personality and stability are widely treasured by researchers in the field of dentistry, as they can be used to predict patients’ perception, treatment modality selection, expectations, compliance and satisfaction with treatment outcome [13]. Subjects with malocclusions might be attributed unfavorable personality traits (e.g. pessimism, anxiety, shyness, self-abasement and introversion), and even presenting difficulties in environment adaptation. It is also confirmed that comparison plays an important role in psychological well-being and that upward comparisons might provoke dysphoric moods [27]. This might be the cause of some high-esteemed people with unpleasant teeth becoming depressed and self-contemptuous.

No definite relationship between personality and psychosocial aspects of esthetics has been established. Spalj et al. found psychosocial outcomes could be well predicted by agreeableness and conscientiousness, while neuroticism, extraversion or openness did not correlate closely [28]. However, it has been reported that psychological factors might influence dental perceptions, which play a significant role in forming satisfaction with dental appearance, and serve to predict their effect on daily living [29]. Moreover, the present result is consistent within prosthodontic restorations [30], where there is a great demand of dental aesthetics too.

In conclusion, our study supports the statement that differences in personality and psychosocial status of young adults are reflected in dental-psychosocial influence. Although perception plays the biggest part in psychosocial impact, the influence of personality factors cannot be neglected. When malocclusion exists, personality builds personal recognition and feelings and/or action towards their own dental appearance.

Contemporary orthodontists increasingly use psychological parameters to assess perceived treatment outcome, moving away from more traditional models that mainly use physical descriptors to characterize success of treatment outcome (such as PAR score reduction) [13]. With psychological assessments, a more comprehensive understanding between public and practitioners can be established and this can in turn benefit the potential subsequent treatments. During clinical treatment, IOTN and varying psychological status should all be factored into consideration so as to achieve personalized therapy and care of their body and mind. Psychological tests and perception status can be used to identify changes to both the occlusion and psychological state. By providing appropriate supportive psychotherapy or counseling at psychological clinics, the adverse effects caused by psychological problem will be significantly reduced during the process. Cognitive behavioral intervention is helpful in challenging dysfunctional body perception for patients with slight clinical treatment need and a high level of psychosocial impairment, who will not improve their psychological condition through orthodontic treatment [19]. This way, the psychological situation improves simultaneously to gradually improve appearance, despite the increase in expenditure. The limitations of increasing costs and lack of orthodontists' psychological assessment ability during clinical procedures need to be dealt with, finding new solutions will be expected for the increasing cost for additional psychological consultancy to patients as well as additional education of psychological assessment for orthodontists.

A patient reported outcome measure (PROM) questionnaire before and after treatment and a patient reported experience measure (PREM) are now widely applied in health care systems. They help assess treatment outcomes of a health condition or disability and the process of healthcare. Outpatient clinics including dental centers should also apply these tools in order to enrich the specialized content and to provide better service to the public.

## Conclusions

Personality plays an important role in psychosocial impact of dental esthetics. Neurotic people will perceive psychosocial impact to a much greater degree, and outgoing people may not be affected psychosocially by dental aesthetics to the same degree. Adequate comprehension about psychosocial health and personality characteristics should be promoted in orthodontics field.

## Data Availability

All data generated or analyzed during this study are included in this published article.

## Abbreviations

EPQ-RSC: Eysenck Personality Questionnaire Short Scale of Chinese version
PIDAQ: Psychosocial Impact of Dental Aesthetics Questionnaire
AC: Aesthetic Component
IOTN: Index of Orthodontic Treatment Need
DHC: Dental Health Component
OHrQol: Oral Health-related Quality of life

## References

[1] Shaw WC, Addy M, Ray C (1980). Dental and social effects of malocclusion and effectivenessof orthodontic treatment: a review. Commun Dent Oral Epidemiol.

[2] Badran SA (2010). The effect of malocclusion and self-perceived aesthetics on the self-esteem of a sample of Jordanian adolescents. Eur J Orthod.

[3] WHOQOL-group. The World Health Organization Quality of Life assessment (WHOQOL): position paper from the World Health Organization. Soc Sci Med (1982). 1995;41(10):1403–9. 10.1016/0277-9536(95)00112-k 8560308

[4] Kumar P, Londhe SM, Kotwal A, Mitra R (2013). Prevalence of malocclusion and orthodontic treatment need in schoolchildren - an epidemiological study. Med J Armed Forces India.

[5] Cardoso CF, Drummond AF, Lages EMB, Pretti H, Ferreira EF, Abreu MHNG (2011). The dental aesthetic index and dental health component of the index of orthodontic treatment need as tools in epidemiological studies. Int J Environ Res Public Health.

[6] Brouns V, de Waal AML, Bronkhorst EM, Kuijpers-Jagtman AM, Ongkosuwito EM (2022). Oral health-related quality of life before, during, and after orthodontic-orthognathic treatment: a systematic review and meta-analysis. Clin Oral Investig.

[7] Kragt L, Dhamo B, Wolvius EB, Ongkosuwito EM (2016). The impact of malocclusions on oral health-related quality of life in children-a systematic review and meta-analysis. Clin Oral Investig.

[8] Klages U, Claus N, Wehrbein H, Zentner A (2006). Development of a questionnaire for assessment of the psychosocial impact of dental aesthetics in young adults. Eur J Orthod.

[9] McManus IC, Furnham A (2006). Aesthetic activities and aesthetic attitudes: influences of education, background and personality on interest and involvement in the arts. Br J Psychol.

[10] Hewitt PL, Sherry SB, Flett GL, Shick R (2003). Perfectionism and cosmetic surgery. Plast Reconst Surg.

[11] Prahl-Andersen B, Boersma H, van der Linden FP, Moore AW (1979). Perceptions of dentofacial morphology by laypersons, general dentists, and orthodontists. J Am Dent Assoc.

[12] Romero-Maroto M, Santos-Puerta N, Gonzalez Olmo MJ, Penacoba-Puente C (2015). The impact of dental appearance and anxiety on self-esteem in adult orthodontic patients. Orthod Craniofac Res.

[13] Zhang L, Liu X, Zheng G-J, Zhou L, Lin D-Y, Wang X-D (2012). Eysenck personality and psychosocial status of adult patients with malocclusion. Asian Pac J Trop Med.

[14] Jiang Q (1998). Primary exploration of comprehensive assessing of psychosocial stress. Chin J Behav Med Sci.

[15] Kerosuo H, Al Enezi S, Kerosuo E, Abdulkarim E (2004). Association between normative and self-perceived orthodontic treatment need among Arab high school students. Am J Orthod Dentofacial Orthop.

[16] Lin H, Quan C, Guo C, Zhou C, Wang Y, Bao B (2013). Translation and validation of the Chinese version of the psychosocial impact of dental aesthetics questionnaire. Eur J Orthod.

[17] Qian M, Wu G, Zhu R, Zhang X (2000). Development of the revised eysenck personality questionnaire short scale for Chinese (EPQ-RSC). Acta Psychol Sin.

[18] Hassan AH, Amin HE-S. Association of orthodontic treatment needs and oral health-related quality of life in young adults. Am J Orthod Dentofacial Orthop. 2010;137(1):42–7. 10.1016/j.ajodo.2008.02.024 20122429

[19] Klages U, Erbe C, Sandru SD, Brullman D, Wehrbein H (2015). Psychosocial impact of dental aesthetics in adolescence: validity and reliability of a questionnaire across age-groups. Qual Life Res.

[20] Afroz S, Rathi S, Rajput G, Rahman SA (2013). Dental esthetics and its impact on psycho-social well-being and dental self confidence: a campus based survey of north Indian university students. J Indian Prosthodont Soc.

[21] Hyde JS (2014). Gender similarities and differences. Annu Rev Psychol.

[22] Solomon D, Katz RV, Bush AC, Farley VK, McGerr TJ, Min H (2016). Psychosocial impact of anterior dental esthetics on periodontal health, dental caries, and oral hygiene practices in young adults. Gen Dent.

[23] Dahong X, Xiangrong C, Ying L, Yusong L, Ying G, Yan S (2013). Effect of incisor position on the self-perceived psychosocial impacts of malocclusion among Chinese young adults. Angle Orthod.

[24] Feu D, de Oliveira BH, de Oliveira Almeida MA, Kiyak HA, Miguel JAM (2010). Oral health-related quality of life and orthodontic treatment seeking. Am J Orthod Dentofacial Orthop.

[25] Lin F, Ren M, Yao L, He Y, Guo J, Ye Q (2016). Psychosocial impact of dental esthetics regulates motivation to seek orthodontic treatment. Am J Orthod Dentofacial Orthop.

[26] Eysenck HJ, Eysenck SBG (1975). Manual of the Eysenck Personality Questionnaire.

[27] Xing S, Yu G (2005). A review on research of social comparison. Adv Psychol Sci.

[28] Spalj S, Novsak A, Bilobrk P, Katic V, Zrinski MT, Pavlic A (2016). Mediation and moderation effect of the big five personality traits on the relationship between self-perceived malocclusion and psychosocial impact of dental esthetics. Angle Orthod.

[29] Karasneh J, Al-Omiri MK, Al-Hamad KQ, Al Quran FAM. Relationship between patients’ oral health-related quality of life, satisfaction with dentition, and personality profiles. J Contemp Dent Pract. 2009;10(6):E049-56. 20020081

[30] Al-Omiri MK, Board J, Karasneh J (2010). Relationship between oral health-related quality of life, satisfaction, and personality in patients with prosthetic rehabilitations. J Prosthodont Implant Esthet Reconst Dent.

